# Stretchable Graphene Thin Film Enabled Yarn Sensors with Tunable Piezoresistivity for Human Motion Monitoring

**DOI:** 10.1038/s41598-019-55262-z

**Published:** 2019-12-09

**Authors:** Mingxuan Bai, Yujiang Zhai, Fu Liu, Yanan Wang, Sida Luo

**Affiliations:** Beihang University, School of Mechanical Engineering & Automation, Beijing, 100191 China

**Keywords:** Sensors and biosensors, Electronic properties and devices

## Abstract

1D graphene based flexible sensors as wearable electronics have recently attracted considerable attentions because of lightweight, high extensibility, easy to wind and weave, and superior sensitivity. In this research, we established a facile and low-cost strategy to construct graphene thin film enabled yarn sensors (GYS) by combining the process of graphene oxide (GO) coating and reducing on polyester (PE) wound spandex yarns. According to systematic processing-property relationship study, a key finding of this work discovers that the degree of resistance recovery as well as gauge sensitivity of GYS can be well controlled and modulated by a pre-stretch treatment. Specifically, as the level of pre-stretch increases from 0 to 60%, the deformable range of sensor that guarantees full resistance recovery prolongs evidently from 0% to ~50%. Meanwhile, the gauge factor of GYS is tunable in the range from 6.40 to 12.06. To understand the pre-stretch process dependent sensing performance, SEM analysis was assisted to evidence the growing size of micro-cracks determining dominantly the behavior of electron transport. Lastly, to take better advantage of GYS, a new wearing mode was demonstrated by direct winding the yarn sensor on varied portions of human body for monitoring different body movements and muscle contracting & relaxing.

## Introduction

Since the last decade, flexible and wearable electronics have been attracting considerable attentions with upgrading concepts and strategies in varied fields of applications, e.g. smart fabrics^1–3^, artificial skins & muscles^4,5^, implantable medical devices^6,7^, man-machine interfaces^8,9^, and internet of things (IoT)^10^. With continuous development, metallic strain gauges as well as other traditional sensing elements/interfaces/devices with mechanical rigidity and/or limited sensitivity are no longer the best candidate to be applied in smart devices^11^. In a sharp contrast, the next-generation sensors enabled by new materials and new nanotechnology are highly demanded to meet the rising standards of high sensitivity, mechanical flexibility, and wearable stretchability^12,13^. Toward this goal, carbon nanomaterials have been widely explored by virtue of their robustness, structural noninvasiveness, and multifunctional properties. Compared with other carbon family members, e.g. fullerene^14^, carbon blacks^15,16^, and carbon nanotubes^17,18^, graphene show multiple unique advantages, including the ease of aqueous colloids preparation, great conformability to substrates and interfaces, and advanced contact-breakage mechanism with excellent piezo-sensitivity, etc.^19–21^. The above-mentioned characteristics have guaranteed a variety of novel strategies for assembling varied types of graphene sensors, e.g., 1D CVD-grown nanofibers^22^, surface-coated fibers^23,24^, hybrid composite fibers^25^, 2D thin films and free-standing papers^26,27^, as well as 3D foams, sponges and aerogels^28^.

When considering embeddable and wearable characteristics for flexible electronics, the sensor with unique one-dimensional format is expected to be more flexible, lightweight, robust, and straightforward to be assembled through winding, weaving, and/or printing processes^19^. Following this line of thought, in combination with extraordinary properties of graphene, different 1D graphene fiber sensors have been proposed for wearable applications. For examples, Wang *et al*. applied CVD method to fabricate graphene/PVA core-sheath nanofibers with the gauge factor of 5.02 and stretchability up to 16.1%^22^. Using one-step wet spinning processes, Jalili *et al*. fabricated reduced graphene oxide fibers with ultimate elongation of ~3% for multifunctional textiles^29^. To meet larger stretching requirements for monitoring human-body movements such as elbow and knee joint actions^30^, graphene enabled composite fibers are demonstrating great advantages when using commercial elastic yarn as the core substrate. By coating thin graphene layers onto elastic fiber yarns through dip-coating and layer-by-layer processes, for instance, Cheng *et al*.^31^, and Park *et al*.^32^ respectively developed graphene covered PE/PU yarns and graphene/PVA coated yarns for monitoring a variety of human motions from head to foot. Montazerian *et al*.^33^ also applied a layer-by-layer process to coat graphene nanoplatelets on silicone sheathed spandex yarns with tunable sensing range >40%. Compared with CVD and wet spinning processed sensors, the composite fibers show better mechanical affinity to woven fabrics, superior tensile performance, and wider applications.

As a new-emergent strategy for assembly of graphene 1D fibers, nevertheless, the sensing performance as well as the mechanism is far from being fully exploited, especially when the commercial yarn core is always specifically-strengthened by multiple sheathed fiber layers twisted/coiled through certain angles. With the overview of relevant works, for one thing, it is still not understandable about the variation of the reported gauge sensitivity varied noticeably from 1.5^34^ to 56.3^35^ with different assembling methods and deformation conditions. For another, it is lacking of a systematic work investigating the structure dependent sensitivity, deformability, and reliability, which is crucial for unveiling the sensing mechanism. With effective capability to alter and control different microscopic features such as micro-cracks^36^, wrinkles^37^, and packing densities^35^, the pre-stretch process has already become a commonly-applied strategy to tune the performance of varied nanocarbon sensors, including processing efficiency^38^, mechanical/electrical stability^39^, as well as sensing sensitivity^37^. Following this line of thought, the application of pre-stretch toward the understanding of process dependent sensing property and mechanism for the unique graphene enabled smart yarn is highly significant. However, the relevant study is still not appeared.

Motivated by the argument mentioned above and to further advance this emerging field, in this research, we systematically explored the unique pre-stretching treatment on regulating both electrical stability and gauge sensitivity toward understanding the sensing behaviors of graphene thin film enabled yarn sensors (GYS). Firstly, a facile and low-cost strategy was established to construct the graphene coating layer on polyester (PE) wound spandex yarns by combining the process of graphene oxide (GO) coating and reducing. Ascribed to the helically covered structure, it is rather interesting that the level of pre-stretch plays an important role on controlling the degree of resistance recovery. As the pre-stretch is increased from 0% to 60%, the ideal deformable range of the sensor that guarantees full resistance recovery prolongs evidently from 0% to ~50%. In addition to the enhanced working range, the pre-stretch dependent sensing performance is highly advisable to optimize the gauge sensitivity of GYS from 6.40 to 12.06. Based on microscopic explorations, the apparent processing-property relationship of GYS is further explained by the variable size of micro-cracks on graphene thin films bridging originally over PE loops, influencing dominantly on both axial and helical directions of electron transport. To take better advantage of lightweight, conformability, tunable sensitivity, and free from packaging medium, a new wearing mode has been demonstrated by direct winding GYS on varied portions of human body for monitoring different motions, such as finger bending and muscle contracting & relaxing.

## Results and Discussion

### Assembly method and structural characterization of smart yarns

**Figure 1:**
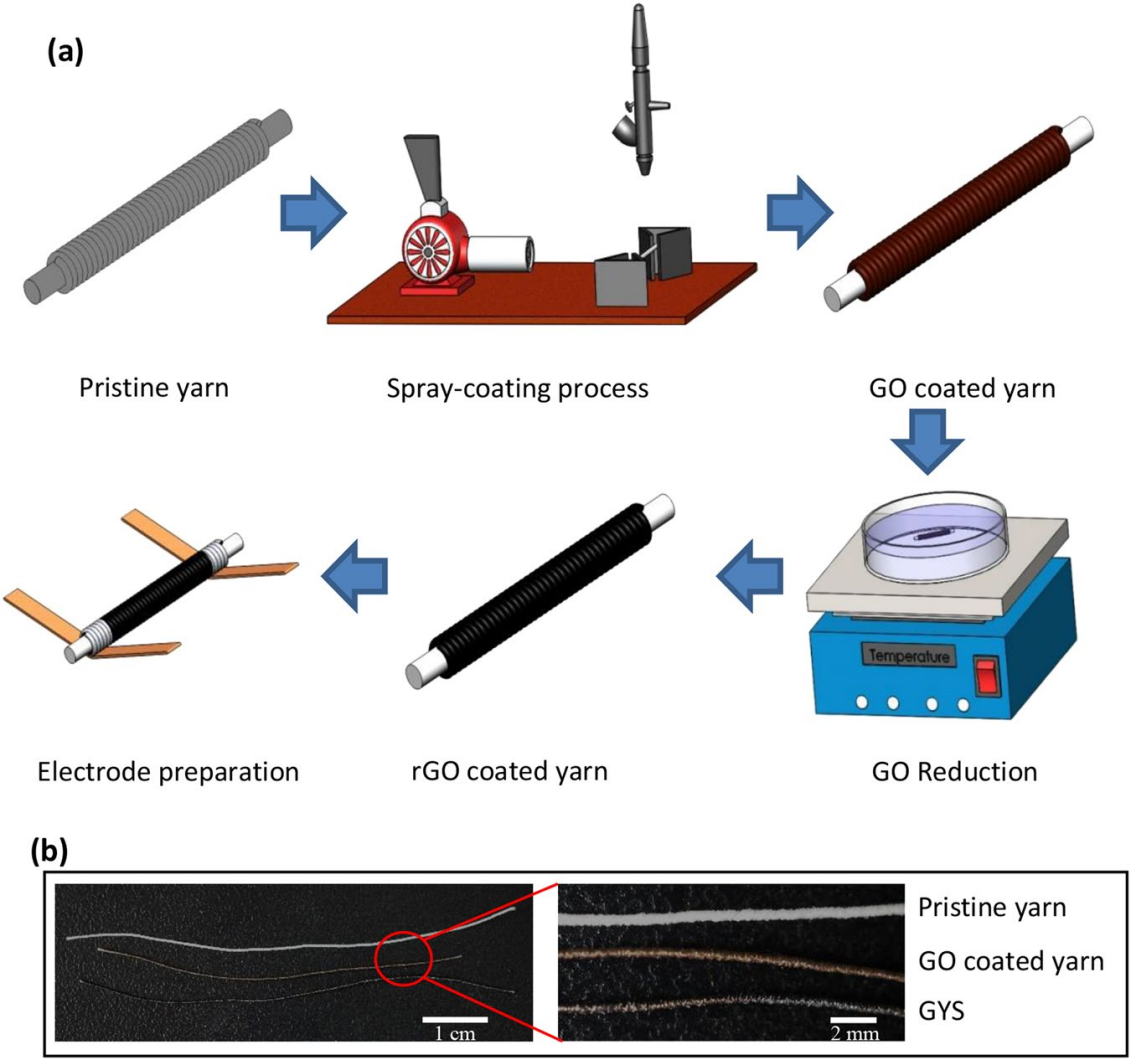
(**a**) Schematic diagram of flow chart showing step-by-step fabrication of the graphene thin film enabled smart yarn (GYS); (**b**) Photographs of the pristine yarn, GO coated yarn and GYS.

Figure 1a schematically shows the processing steps for fabrication of graphene thin film enabled yarn sensors (GYS), including spray-coating of GO thin film, chemical reduction of GO, and electrode wire connection. Assisted by computer controlled moving stage, the spray-coating becomes a precise and high-efficient process for thin-film formation through depositing the mist of GO dispersion (10 mL) onto the polymer yarn, with controlled nozzle-to-sample height (6 cm), back-and-forth moving speed (2 cm/s), spraying rate (2.5 mL/min), and pre-heated temperature (100 °C). Based on visual, photographical and microscopic inspections, the effective GO coating has been proved by coloring the pristine yarn from ivory white to yellowish brown (Fig. 1b). Following spraying process, the GO coated yarn was immersed in 90 °C pre-heated 0.06 mmol/L sodium hydrosulfite solution for 150 min for achieving GYS with reduced graphene oxide (rGO) coating layer. Instead of using hydroiodic acid with destructive effect on polyester fibers^40^, the Na_2_S_2_O_4_ solution is applicable by oxidizing S_2_O_4_^2−^ to SO_3_^2−^ through reacting with hydroxyl and epoxide groups in GO^40,41^. Once again, the effective reducing process was convincingly proved by the pitch-black appearance of GYS as shown in Fig. 1b and Supplementary Fig. S1. Lastly, with 100 °C heating treatment for intermediate product elimination (e.g. NaHSO_3_) as well as copper electrode connection, the as-produced GYS sensors were ready for the following structural analysis and performance evaluation.

**Figure 2:**
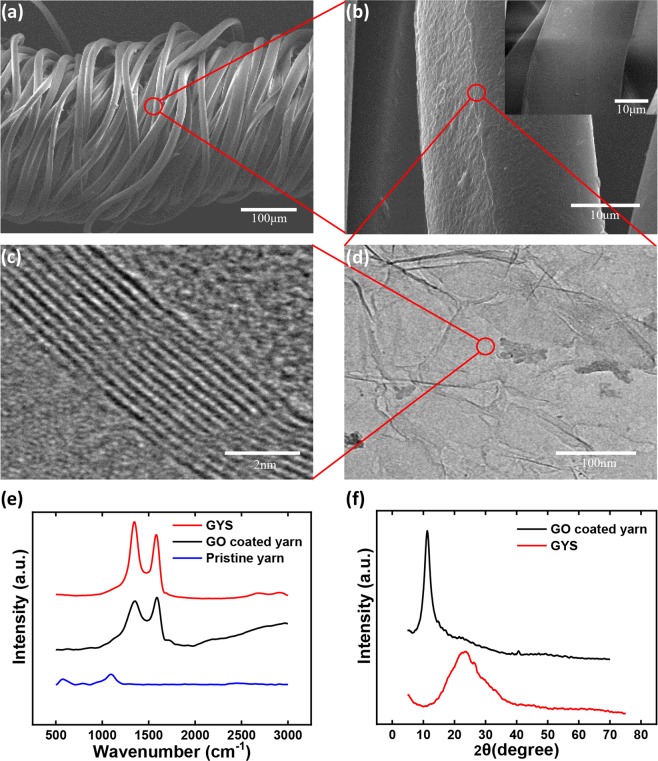
(**a,b**) SEM images of GYS and pristine yarn shown in the inset plot of (**b**); (**c,d**) TEM images showing detailed structures of GYS; (**e**) Raman and (**f**) XRD spectra for comparison of pristine yarn, GO coated yarn, and GYS.

In addition to visual inspection, structural and chemical details of GYS were further evaluated by varied microscopic and spectroscopic methods. Low-magnified scanning electron microscope (SEM) image (Fig. 2a) first unveils the sheath-core structure of the composite yarn, constituting the spandex inner core and the PE fiber jacket winded helically onto the inner fiber surface. The utilization of the spring-like sheath-core architecture for wearable device assembly is critical for boosting the sensing performance^31^. Under higher magnification, Fig. 2b reveals the graphene thin layer coated on the helical fiber, showing clear contrast to the pristine PE with smooth surface (inset picture). The apparent ripple-like wrinkled structure of graphene along with the few-layered geometry is further confirmed by transmission electron microscope (TEM) images, as shown in Fig. 2c and d. To achieve more detailed structural information, Raman and X-ray Diffraction (XRD) analyses were examined at different assembling cycles of the sensor device, including pristine yarn, GO coated yarn, and the as-produced GYS. As compared with the featureless spectrum of the neat yarn, Fig. 2e shows prominent Raman D and G peaks locating respectively around 1353 cm^−1^ and 1582 cm^−1^ for both GO and GYS, confirming the existence of graphitic structures. After the process of GO reduction, the increased intensity ratio of D and G peak (I_D_/I_G_) from 0.968 to 1.072 suggests both the decrease in average size of the sp^2^ domain as well as the increased quantity of structural defects^42^. In addition to Raman, XRD spectra show clearly the shift of the characteristic 2θ peak from 11.38° to 24.27° after the reduction process, reflecting the removal of oxygen-containing groups and the decrease of interlayer spacing from 0.778 nm to 0.360 nm which is fairly close to the one acquired from TEM (0.350 nm)^43^.

### Tunable piezoresistive properties of smart yarns

**Figure 3:**
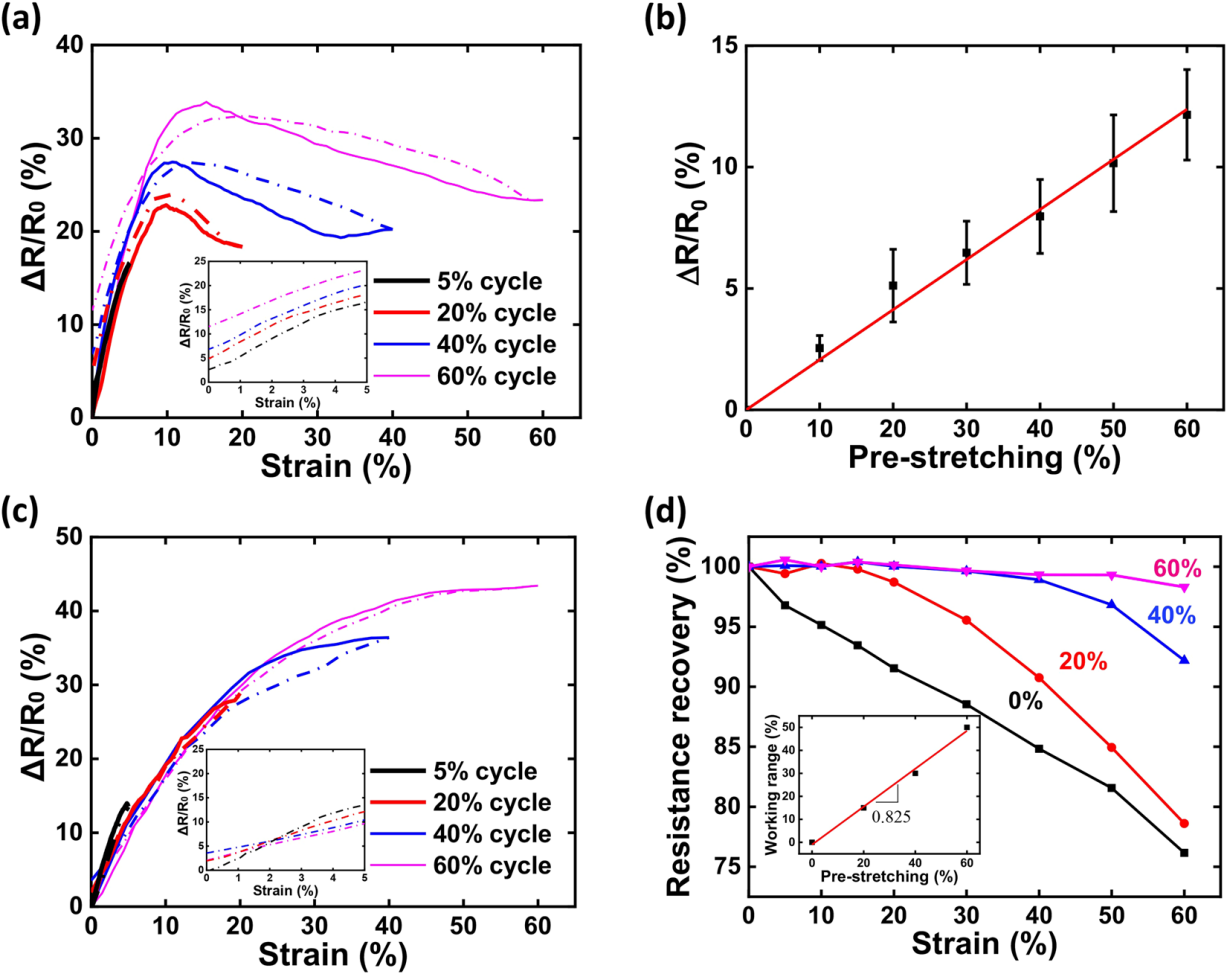
(**a**) Real-time resistance change of GYS sensors subjected to single-cycle tensile test with varied strain ranges, including 5%, 20%, 40% and 60%; (**b**) The resistance variation of GYS subjected to different levels of pre-stretching processes; (**c**) Real-time resistance change of varied pre-stretch treated GYS sensors subjected to single-cycle tensile test with varied strain ranges; (**d**) Strain dependent recovery degree of resistance of GYS sensors under varied pre-stretch treatments; Inset plots of (**a**) and (**c**) show the detailed releasing stages of the resistance change; Inset plot of (**d**) shows the linear increment of working range as the function of pre-stretch.

In order to systematically evaluate the sensing property, coupled electrical - cyclic tensile tests were applied to acquire piezoresistive responses of GYS sensors^44,45^. Through the measurement, sensitivity of the smart yarn can be quantified by determining the gauge factor (GF), which is defined as d(ΔR/R_0_)/dε where ε is the mechanical strain applied to the yarn, R_0_ and R are respectively the resistance of GYS before and after deformation. Since the length of the yarn cannot be completely restored when mechanical strain exceeds 60% ascribed to its viscoelastic behavior^46^, the upper limit of its working range is bound to this critical value for the ease of cyclic testing. Figure 3a as well as the inset plot first records the resistance variation of GYS sensors subjected to the single-cycle tensile test with varied strain ranges. During the stage of stretching (solid lines), the resistance change (ΔR/R_0_) accordingly varies as the enhancement of mechanical deformation from 0 to 5/20/40/60%, intriguing the piezoresistive behavior of the conducting layer. During the stage of releasing (dashed lines), nevertheless, the decreasing trend of ΔR/R_0_ is not able to follow its rising trace. More seriously, the resistance fails to recover back to its initial value regardless of the level of the subjected deformation. The inset picture also clears that the higher the degree of stretching, the greater is the resistance when totally relaxed with the zero-strain ΔR/R_0_ ranging from 2.6% (5% strain treated) to 11.5% (60% strain treated). To better quantify this behavior, a representative GYS specimen as produced by the spraying & reducing assembly was successively treated by different pre-stretching processes from low to high levels. As the pre-stretch level is steadily increased from 0 to 60%, Fig. 3b obviously unveils a linear increase of the change in initial resistance from 0% to 12%. The observed increase of initial resistance is a clear indication of permanent changes of the graphitic conducting network when the helical fiber substrate is deformed to certain stages. Obviously, the above issue is requisite to be resolved which may further hamper the real application for wearable activities.

Benefit from the cyclic test, it is worth noting that the pre-stretching process plays a dominant effect on recovery of initial resistance. It means that if a GYS sensor has experienced certain treatment of mechanical stretching, the irreversible issue of resistance could be weakened in next stretching cycles. To prove the idea, a comparison study was examined for investigating the behavior of strain dependent resistance change (ΔR/R_0_) after experiencing the corresponding treatment of pre-stretch. As shown in Fig. 3c and its inset, for each pre-stretched tensile cycle, not only the tensing & releasing traces can be basically reproduced, the final resistance is also capable of returning back to the initial level (<4%). The above result indicates clearly that the effect of permanent structural changes of GYS can be substantially reduced by the pre-stretch treatment. To further evidence this important clue, systematic analysis has been explored for mapping the recovery level of resistance as the function of mechanical strain under varied pre-stretching conditions. Based on the definition – (R_max_ − R_f_)/(R_max_ − R_0_) where R_max_, R_f_, and R_0_ are respectively maximum, final, and initial resistance, Fig. 3d unveils clearly the result. Without the pre-stretch treatment, the recovery degree decays immediately from 100% to 78.6% as the increase of deformation up to 60%. With certain level of pre-stretch, the 100% recovery of resistance could keep sustained before the deformation exceeds a critical value. By considering the critical value as the new working range of GYS sensors, the inset plot of Fig. 3d shows the linear increment of working range (0–~50%) as the function of the pre-stretch level (0–60%), in which the slope equals to 0.825. With the combined results in Fig. 3c,d, the determined pre-stretch dependent working range could further represent the sensing range of various GYS sensors, showing continuous and reversible electrical properties.

**Figure 4:**
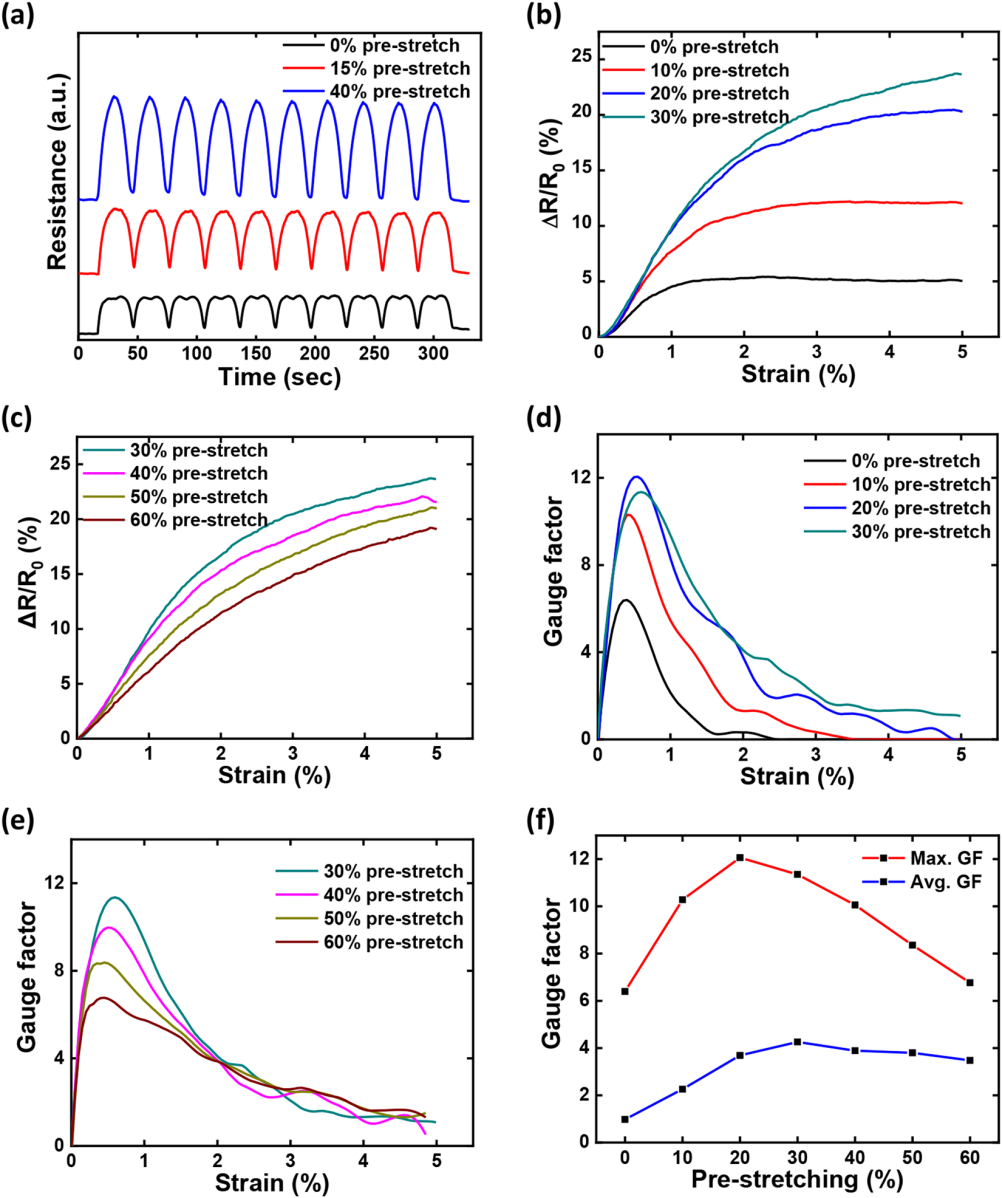
(**a**) 10 cycled resistance variation of three GYS sensors subjected to different pre-stretch conditions; (**b,c**) Strain dependent resistance change of GYS sensors prepared under varied pre-stretch conditions; (**d,e**) Strain dependent gauge factor of GYS sensors prepared under varied pre-stretch conditions; (**f**) Maximized and averaged gauge factors of GYS sensors prepared under varied pre-stretch conditions.

In addition to improve the working range, the pre-stretch treatment also plays an important role on controlling and modulating the sensing properties of varied GYS sensors. Figure 4a displays and compares the 10 cycled electrical-tensile performance by constantly stretching and releasing the GYS with different pre-stretch conditions. When no pre-stretch is applied, the resistance swiftly reaches to a plateau value when strain is higher than 2.4%, showing clearly that the resistance change is only controlled by a rather limited strain range. With 15% or 40% pre-stretch, continuous increase and decrease of the resistance can be observed. When higher pre-stretch level is applied, the resistance ramps faster, indicating the strain-sensing performance with higher sensitivity. Based on the fundamental analysis, systematic results have been summarized for comparing the sensing performance of the variedly treated GYS. Figure 4b and c first show the feature of resistance change with the pre-stretch ranging from 0% to 60%. When the level is smaller than 30%, Fig. 4b confirms the previously suggested conclusion that the higher pre-stretch introduces both faster resistance ramping and wider strain range for resistance increase. Nevertheless, when the treatment exceeds 30%, negative effect has been observed. As shown in Fig. 4c, higher leveled pre-stretch determines slower resistance ramping. By converting ΔR/R_0_ to GF according to its definition mentioned previously, gauge sensitivity of varied GYS sensors is further summarized. The results as shown in Fig. 4d and e once again evidence the process dependent characteristics. For all processing conditions, the dynamic GFs behave similarly so that it increases promptly to the maximum for the first ~0.5% strain and then is followed by a decaying tendency. However, the absolute sensitivity is varied markedly from one to another. For examples, the one without the pre-stretch is showing the maximum GF of 6.4 at ~0.38% and totally losing its piezoresistive capability around 2.4%, while the one with 30% pre-stretch possesses much improved sensitivity (GF = 11.4) at ~0.6% and still shows effective sensing behavior (GF = 1.1) at 5%. Supplementary Figs. S2, S3 further discusses the linearity and long-term durability of piezoresistive properties, equivalently evidencing the prestretch dependent behavior. To better quantify the sensing property, Fig. 4f respectively summarizes the relationship between the pre-stretch condition and the maximum/averaged gauge sensitivity. It is rather advisable that to produce GYS sensors with higher piezoresistive sensitivity, a moderate pre-stretch process needs to be applied in the range between ~20% and ~40%.

**Figure 5:**
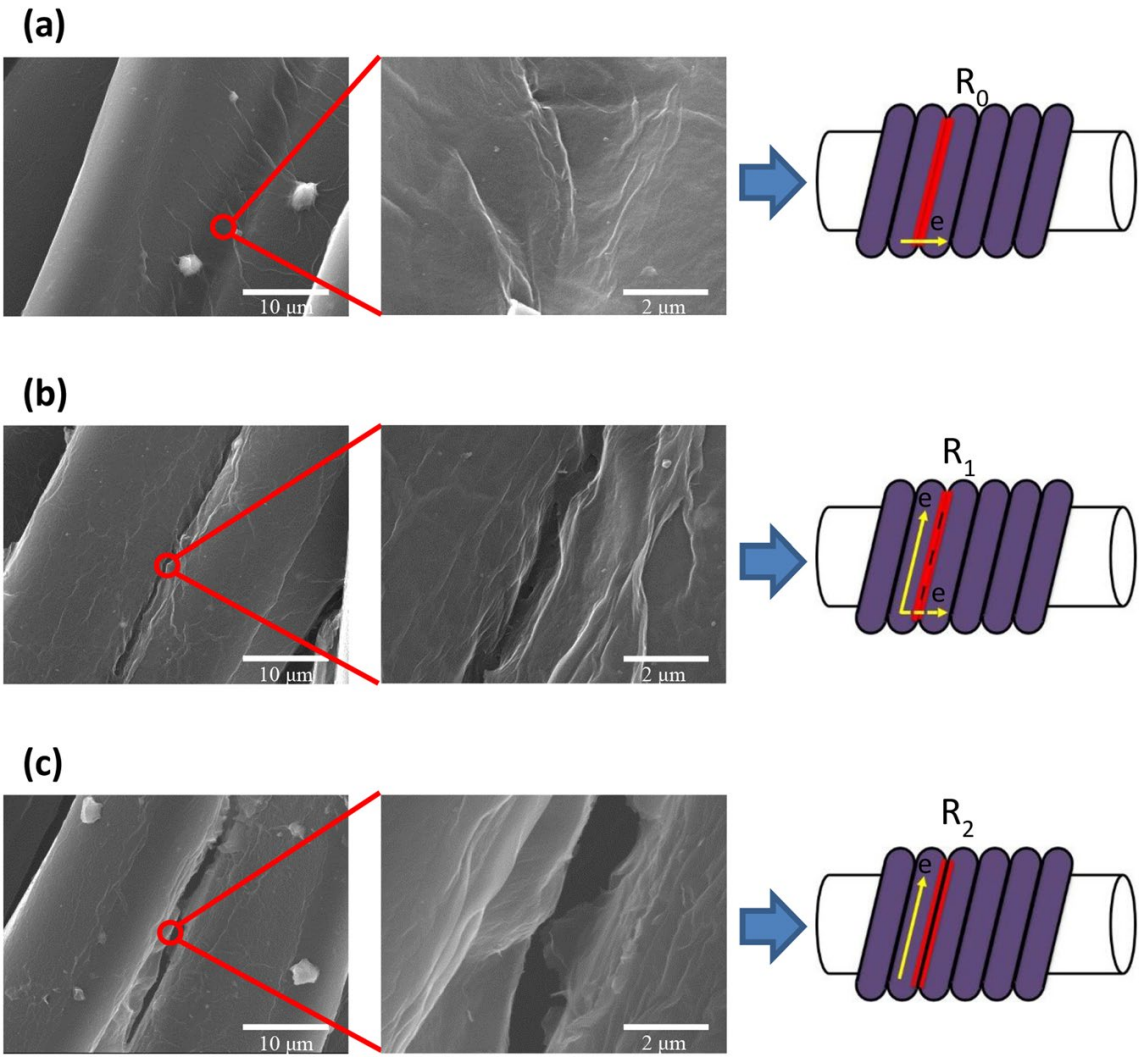
SEM images and schematic diagrams of contact resistance of adjacent PE fibers in three cases: the adjacent PE fibers are coated with (**a**) smooth graphene films; (**b**) graphene films with micro-cracks; (**c**) graphene films with larger cracks.

To understand the sensing mechanism, SEM images were further assisted to speculate the electron transport behavior in different GYS sensors. Without pre-stretch, the microscopic pictures in Fig. 5a show clearly that the graphene layer is able to cover across the two adjacent winding loops. As a result, the electron reasonably transports along the axial direction of the polymer yarn where the graphene coating is complete as a bridge. In this situation, the strain dependent resistance change is not sensitive because of the integrity of the conducting network. When moderate level of pre-stretch (<40%) has been applied, micro-cracks can be observed in Fig. 5b. It is believed that the pre-stretch process mildly slits the thin film when the looped fiber is tensioned. The existence of the tiny fissure could partially block the electron from transporting axially. Therefore, the gauge behavior becomes sensitive because of the increased contact resistance when the crack is enlarged under tension. If processed by a large pre-stretch (>40%), the generated crack is big enough to isolate the adjacent fiber loops so most of electrons would transport around the loop instead of moving axially (Fig. 5c). In this situation, the gauge sensitivity is reduced again. Based on unique graphene coated sheath-core structure as well as SEM observation, the competing of the two coupled axially & helically transporting modes of electron thus dominates the observed non-monotonic change of piezoresistivity with the increased level of pre-stretch.

### Motion monitoring applications of smart yarns

**Figure 6:**
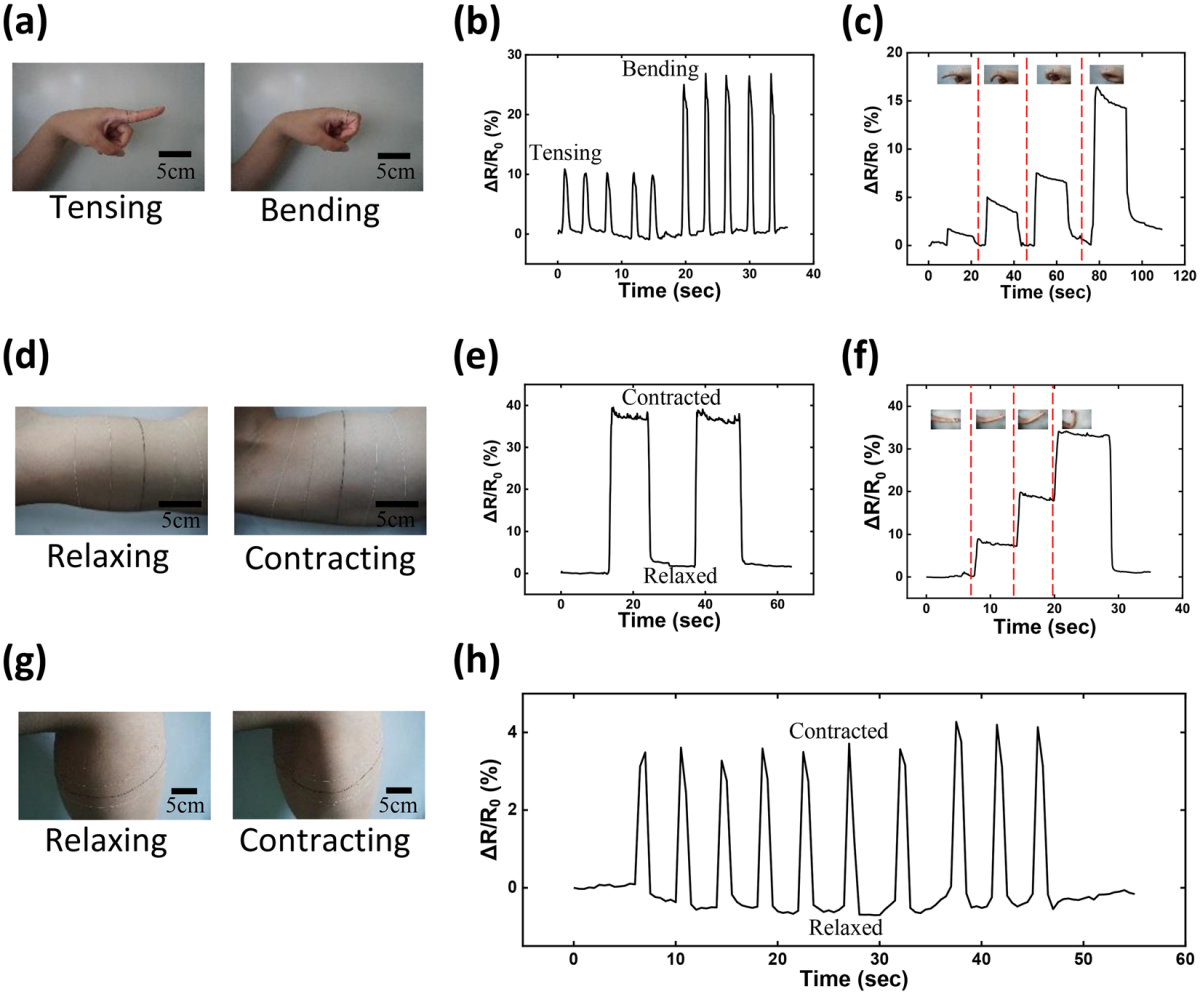
Photographs of GYS sensors wrapped around (**a**) a finger, (**d**) an upper arm, and (**g**) a lower leg; A series of motion monitoring signals with specific resistance waveforms, including (**b**) instant tensing, continuous bending and (**c**) static motions of a finger, (**e**,**f**) varied contractions of biceps through elbow movement, and (**h**) repeated contracting & relaxing of the lower leg muscle.

In comparison to other carbon based piezoresistive sensors, e.g. carbon nanotube or graphene thin films^47,48^, one of the unique features of GYS sensors is their 1D geometry with large extensibility. This unique property in combination with their reversible conductivity, high gauge sensitivity, wash durability (Supplementary Fig. S4), as well as lightweight and package-free configuration makes the GYS highly promising for applications in human motion monitoring. To take better advantage of the above-mentioned structural and physical characteristics, a new wearing mode of the flexible sensing device has been demonstrated by direct winding the GYS on varied portions of the human body without additional processes such as substrate attaching and textile braiding. The direct wearing mode could also arouse the enhancement of sensing accuracy because no extra interfacial medium has been introduced. As displayed in varied photographs of Fig. 6, the 30% pre-stretch treated GYS sensors with accordingly appropriate length were wrapped around finger, upper arm, and lower leg of a human body. With conformal movement of joints or tension/relaxation of muscle tissues, different types of human motions can be faithfully registered by the real-time resistance change of the yarn sensors ascribing to their high flexibility, extensibility and sensitivity. To demonstrate this, Fig. 6 records a series of the motion monitoring signals with specific resistance waveforms, including instant tensing (left part of Fig. 6b), continuous bending (right part of Fig. 6b) and static motions (Fig. 6c) of a finger, varied contractions of biceps through elbow movement (Fig. 6e,f), and repeated contracting & relaxing of the lower leg muscle (Fig. 6h). A Supplementary video (Video S1) is also assisted to restore the varied motion monitoring applications. To further evidence the superiority, Supplementary Fig. S5 compares the direct winding mode with other two frequently applied wearing methods, i.e., PDMS packaging and fabric/gauze braiding, showing higher sensitivity, faster response, and better electrical stability. According to the successful demonstrations, we highly anticipate the in-depth utilization of the GYS technology for a broad range of applications in motion monitoring and man-machine interactions, e.g. remotely control of robotic arm or specialized vehicle based on human gestures, rehabilitation progress monitoring of a patient with different respiratory and blood pressure conditions, and athlete training and fatigue monitoring for injury avoidance and performance boosting.

## Conclusion

In summary, we demonstrated a facile and low-cost strategy to construct graphene thin film enabled yarns (GYS) by combining the process of graphene oxide (GO) coating and reducing on polyester (PE) wound spandex yarns. The effect of pre-stretch processes on sensing sensitivity and degree of resistance recovery of GYS was studied systematically. The research results show that proper pre-stretch can optimize the performance of GYS. The ideal deformable range of the sensor that guarantees full resistance recovery increases from 0% to 50% after applying a 60% pre-stretch. As for the sensing performance, the gauge sensitivity increases from 6.40 to 12.06 when applied by a 30% pre-stretch. The sensing mechanism was further explained by exploring the micromorphology. With the augmentation of pre-stretch levels, the size of micro-cracks on graphene thin film grows accordingly, introducing the change of electron transport behavior on graphene thin films. As contact resistance increases, GYS becomes more sensitive but shows negative effect when beyond a critical value, ascribing to the cut off of the axial transmission path of electrons. Based on fundamental study, a new wearing mode of the yarn sensor by direct winding GYS on varied portions of human body has been demonstrated for monitoring different motions, e.g. finger bending and muscle contracting. The unique characteristics of GYS sensors with lightweight, robust, tunable sensitivity, and free from packaging medium demonstrated in this work can be highly valuable for developing next-generation wearable electronics.

## Methods

### Fabrication process of GYS

The graphene oxide (GO) dispersion was prepared by sonicating a mixture of 600 mg of graphene oxide nanosheets which was synthesized via the Hummers method in 200 mL of deionized water in an ice bath using an ultrasonic processor (Ultrasonics FS-600 N). The ultrasonic processor was operated in a pulse operation mode (on 10 s, off 10 s) with the power set at 510 W for 3 h to obtain the 3 mg/mL GO dispersion.

The spandex yarns wound around polyester (PE) are commercially available. The yarn with the cut length of 5.5 cm was cleaned repeatedly using deionized water and dried with a heat gun for 5 min. A pair of clamps was used to fix the yarn sitting on the platform of the spray-coating system, consisting of a heat gun, an air compressor (Iwata IS-50), and an air brush (Iwata HP-CS) equipped on a computer controlled moving stage (Nordson E3). The heat gun was used to accelerate solvent evaporation and the air compressor was used to provide the pressure needed for spraying. In view of the requirement to avoid the heat gun wind for disrupting the spraying process, the heating machine was placed perpendicular to the air brush. To control the heating temperature of the sample around 100 °C, the distance from the heat gun to the yarn is set at 18.5 cm. Additionally, the distance from the nozzle to the elastic substrate is about 6 cm for best spraying effect. Before spraying, the yarn was slightly stretched to prevent it from shaking violently during the coating process.

With the back-and-forth nozzle moving speed of 2 cm/s and spraying rate of 2.5 mL/min, 5 mL as-prepared GO dispersion was sprayed on the yarn surface with the accompanied color change from white to brown. After the top-direction spraying was completed, the yarn was axially rotated by 180° and received another 5 mL spraying treatment to ensure the uniform coating of graphene oxide. Finally, the GO coated yarn was soaked in 90 °C pre-heated 0.06 mmol/L sodium hydrosulfite solution for 2 h and 30 min. The reduced GO (rGO) yarn was washed with deionized water for several times and dried before use.

### Structural characterization and performance evaluation

The SEM characterization was accomplished using a JEOL JSM-7001F FE-SEM. Raman spectra were recorded on a HORIBA LabRAM HR Evolution spectrometer. XRD characterization was performed on Rigaku D/max 2550 with Cu Kα radiation (λ = 1.54°). In addition, the yarn was sputter coated with platinum before being examined by FE-FEM.

To facilitate measurement and reduce contact resistance, two copper pieces were pasted onto each end of the yarn using conductive silver paste before strain sensing tests. The electric properties of the smart yarns were measured by a Keithley 2510 source meter. The sensing performances were evaluated by a mechanical-electric coupling test including a universal testing machine (MTS E44.104 with 500 N load cell) and the source meter. The tension was controlled by the universal testing machine with the fixed deformation rate of 20 mm/min and real-time changes of the sensor resistance were recorded by the source meter programmed by LabVIEW. The sensing performance under other deformation rates was summarized in Supplementary Fig. S6, showing no frequency dependence.

## Supplementary information

Video S1

Supplementary Document

## References

[1] Karim N (2017). Scalable Production of Graphene-Based Wearable E-Textiles. ACS Nano.

[2] Luo S (2017). CNT Enabled Co-braided Smart Fabrics: A New Route for Non-invasive, Highly Sensitive & Large-area Monitoring of Composites. Scientific Reports.

[3] Ma Z, Xu R, Wang W, Yu D (2019). A wearable, anti-bacterial strain sensor prepared by silver plated cotton/spandex blended fabric for human motion monitoring. Colloids and Surfaces A: Physicochemical and Engineering Aspects.

[4] Yang T (2015). Tactile Sensing System Based on Arrays of Graphene Woven Microfabrics: Electromechanical Behavior and Electronic Skin Application. ACS Nano.

[5] Chun S (2015). A tactile sensor using a graphene film formed by the reduced graphene oxide flakes and its detection of surface morphology. Carbon.

[6] Tao LQ (2017). Graphene-Paper Pressure Sensor for Detecting Human Motions. ACS Nano.

[7] Gong S (2014). A wearable and highly sensitive pressure sensor with ultrathin gold nanowires. Nature Communications.

[8] Luo S, Liu T (2013). SWCNT/graphite Nanoplatelet Hybrid Thin Films for Self-temperature-compensated, Highly Sensitive, and Extensible Piezoresistive Sensors. Advanced Materials.

[9] Gong S (2015). Tattoolike Polyaniline Microparticle-Doped Gold Nanowire Patches as Highly Durable Wearable Sensors. ACS Applied Materials & Interfaces.

[10] Liu G (2018). A Flexible Temperature Sensor Based on Reduced Graphene Oxide for Robot Skin Used in Internet of Things. Sensors.

[11] Tung TT (2017). Recent Advances in Sensing Applications of Graphene Assemblies and Their Composites. Advanced Functional Materials.

[12] Wang F, Liu S, Shu L, Tao XM (2017). Low-dimensional carbon based sensors and sensing network for wearable health and environmental monitoring. Carbon.

[13] Yang T, Xie D, Li Z, Zhu H (2015). Recent advances in wearable tactile sensors: Materials, sensing mechanisms, and device performance. Materials Science and Engineering: R: Reports.

[14] Shi, X., Liu, S., Sun, Y., Liang, J. & Chen, Y. Lowering Internal Friction of 0D-1D-2D Ternary Nanocomposite-Based Strain Sensor by Fullerene to Boost the Sensing Performance. *Advanced Functional Materials* **28** (2018).

[15] Ke K, Potschke P, Wiegand N, Krause B, Voit B (2016). Tuning the Network Structure in Poly(vinylidene fluoride)/Carbon Nanotube Nanocomposites Using Carbon Black: Toward Improvements of Conductivity and Piezoresistive Sensitivity. ACS Applied Materials & Interfaces.

[16] Wu X, Han Y, Zhang X, Zhou Z, Lu C (2016). Large-Area Compliant, Low-Cost, and Versatile Pressure-Sensing Platform Based on Microcrack-Designed Carbon Black@Polyurethane Sponge for Human-Machine Interfacing. Advanced Functional Materials.

[17] Luo S, Obitayo W, Liu T (2014). SWCNT-thin-film-enabled fiber sensors for lifelong structural health monitoring of polymeric composites - from manufacturing to utilization to failure. Carbon.

[18] Li Y, Luo S, Yang M, Liang R, Zeng C (2016). Poisson ratio and piezoresistive sensing; a new route to high-performance 3D flexible and stretchable sensors of multimodal sensing capability. Advanced Functional Materials.

[19] Geim AK, Novoselov KS (2007). The rise of graphene. Nature Materials.

[20] Wang G (2018). Structure dependent properties of carbon nanomaterials enabled fiber sensors for *in situ* monitoring of composites. Composite Structures.

[21] Luo S, Hoang PT, Liu T (2016). Direct laser writing for creating porous graphitic structures and their use for flexible and highly sensitive sensor and sensor arrays. Carbon.

[22] Wang X, Qiu Y, Cao W, Hu P (2015). Highly stretchable and conductive core-sheath chemical vapor deposition graphene fibers and their applications in safe strain sensors. Chemistry of Materials.

[23] Luo S, Wang G, Wang Y, Xu Y, Luo Y (2019). Carbon nanomaterials enabled fiber sensors: A structure-oriented strategy for highly sensitive and versatile *in situ* monitoring of composite curing process. Composites Part B: Engineering.

[24] Luo S, Liu T (2014). Graphite nanoplatelet enabled embeddable fiber sensor for *in situ* curing monitoring and structural health monitoring of polymeric composites. ACS Applied Materials & Interfaces..

[25] Cao J, Wang C (2018). Highly conductive and flexible silk fabric via electrostatic self assemble between reduced graphene oxide and polyaniline. Organic Electronics.

[26] Wang Y (2019). Freestanding laser induced graphene paper based liquid sensors. Carbon.

[27] Wang Y, Wang Y, Zhang P, Liu F, Luo S (2018). Laser-induced freestanding graphene papers: a new route of scalable fabrication with tunable morphologies and properties for multifunctional devices and structures. Small.

[28] Liu H (2017). Lightweight conductive graphene/thermoplastic polyurethane foams with ultrahigh compressibility for piezoresistive sensing. Journal of Materials Chemistry C.

[29] Jalili R (2013). Scalable One-Step Wet-Spinning of Graphene Fibers and Yarns from Liquid Crystalline Dispersions of Graphene Oxide: Towards Multifunctional Textiles. Advanced Functional Materials.

[30] Yamada T (2011). A stretchable carbon nanotube strain sensor for human-motion detection. Nature Nanotechnology.

[31] Cheng Y, Wang R, Sun J, Gao L (2015). A stretchable and highly sensitive graphene-based fiber for sensing tensile strain, bending, and torsion. Advanced Materials.

[32] Park JJ, Hyun WJ, Mun SC, Park YT, Park OO (2015). Highly stretchable and wearable graphene strain sensors with controllable sensitivity for human motion monitoring. ACS Applied Materials & Interfaces.

[33] Montazerian H (2019). Graphene-Coated Spandex Sensors Embedded into Silicone Sheath for Composites Health Monitoring and Wearable Applications. Small.

[34] Souri H, Bhattacharyya D (2018). Wearable strain sensors based on electrically conductive natural fiber yarns. Materials & Design.

[35] Son W (2019). Ecoflex-Passivated Graphene-Yarn Composite for a Highly Conductive and Stretchable Strain Sensor. J Nanosci Nanotechnol.

[36] Liu Q, Chen J, Li Y, Shi G (2016). High-Performance Strain Sensors with Fish-Scale-Like Graphene-Sensing Layers for Full-Range Detection of Human Motions. ACS Nano.

[37] Leem Juyoung, Lee Yeageun, Wang Michael Cai, Kim Jin Myung, Mun Jihun, Haque Md Farhadul, Kang Sang-Woo, Nam SungWoo (2019). Crack-assisted, localized deformation of van der Waals materials for enhanced strain confinement. 2D Materials.

[38] Yan T, Wang Z, Pan Z-J (2018). A highly sensitive strain sensor based on a carbonized polyacrylonitrile nanofiber woven fabric. Journal of Materials Science.

[39] Lu Y (2018). High-Performance Stretchable Conductive Composite Fibers from Surface-Modified Silver Nanowires and Thermoplastic Polyurethane by Wet Spinning. ACS Applied Materials & Interfaces.

[40] Kuila T (2012). Chemical functionalization of graphene and its applications. Nature Nanotechnology.

[41] Zhou T (2011). A simple and efficient method to prepare graphene by reduction of graphite oxide with sodium hydrosulfite. Nanotechnology.

[42] Beams R, Gustavo Cancado L, Novotny L (2015). Raman characterization of defects and dopants in graphene. Journal of Physics: Condensed Matter.

[43] Stobinski L (2014). Graphene oxide and reduced graphene oxide studied by the XRD, TEM and electron spectroscopy methods. Journal of Electron Spectroscopy and Related Phenomena.

[44] Luo S (2018). Hybrid spray-coating, laser-scribing and ink-dispensing of graphene sensors/arrays with tunable piezoresistivity for *in situ* monitoring of composites. Carbon.

[45] Luo S, Liu T (2013). Structure-property-processing Relationships of Single-wall Carbon Nanotube Thin Film Piezoresistive Sensors. Carbon.

[46] Babay A, Helali H, Msahli S (2014). Study of the mechanical behaviour of the elastic-core-spun yarns. Journal of the Textile Institute Proceedings & Abstracts.

[47] Liu H (2016). Piezoresistive behavior of porous carbon nanotube-thermoplastic polyurethane conductive nanocomposites with ultrahigh compressibility. Applied Physics Letters.

[48] Smith AD (2016). Piezoresistive properties of suspended graphene membranes under uniaxial and biaxial strain in nanoelectromechanical pressure sensors. ACS Nano.

