# Supplementary material for: Stretchable Graphene Thin Film Enabled Yarn Sensors with Tunable Piezoresistivity for Human Motion Monitoring
Source: Sci Rep. 2019 Dec 9;9:18644. doi: 10.1038/s41598-019-55262-z (PMC6901454; doi:10.1038/s41598-019-55262-z)
Supplement: Supplementary file 2 — Supplementary Document [file 41598_2019_55262_MOESM2_ESM.docx]

Stretchable Graphene Thin Film Enabled Yarn Sensors with Tunable Piezoresistivity for Human Motion Monitoring

Mingxuan Bai^1,#^, Yujiang Zhai^1,#^, Fu Liu^1^, Yanan Wang^1^ and Sida Luo^1,^*

^1^Beihang University, School of Mechanical Engineering & Automation, Beijing, 100191, China

[*s.luo@buaa.edu.cn](mailto:*s.luo@buaa.edu.cn)

^#^Mingxuan Bai and Yujiang Zhai contributed equally to this work

**Supplementary Information**


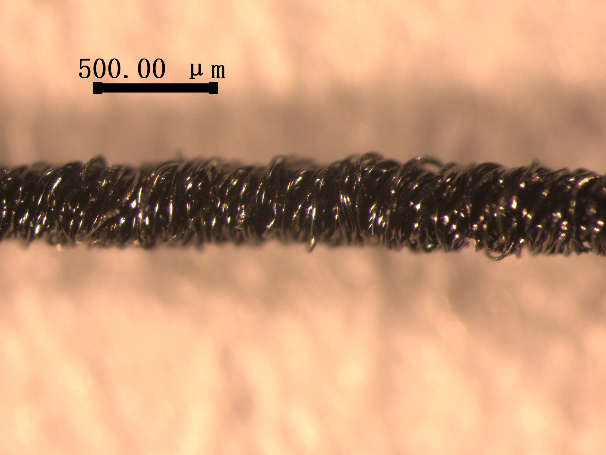

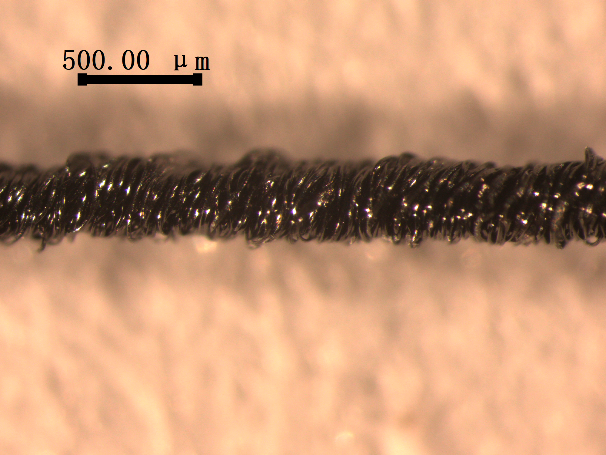

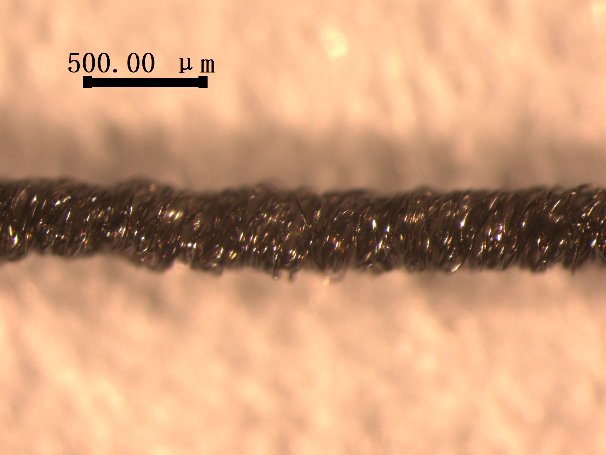

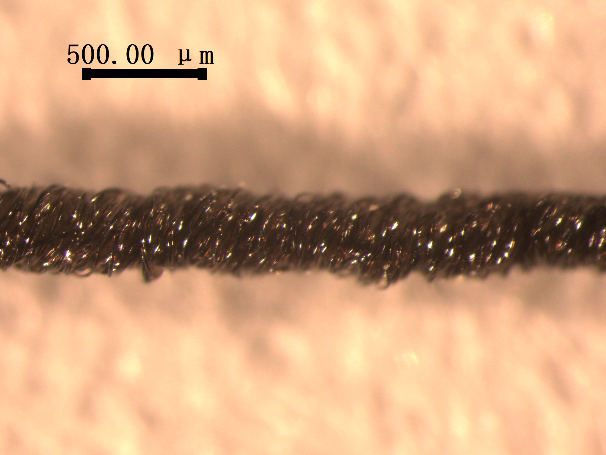

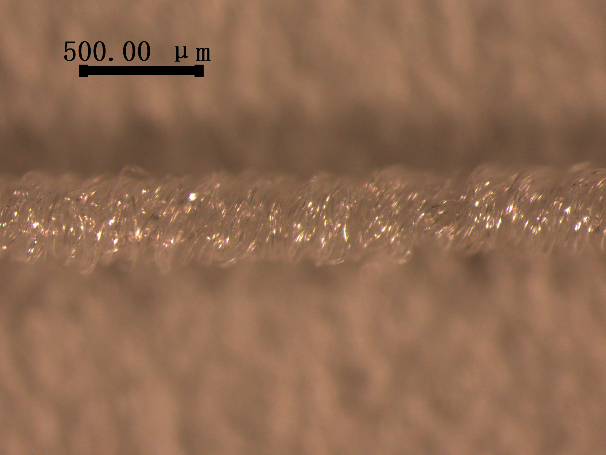

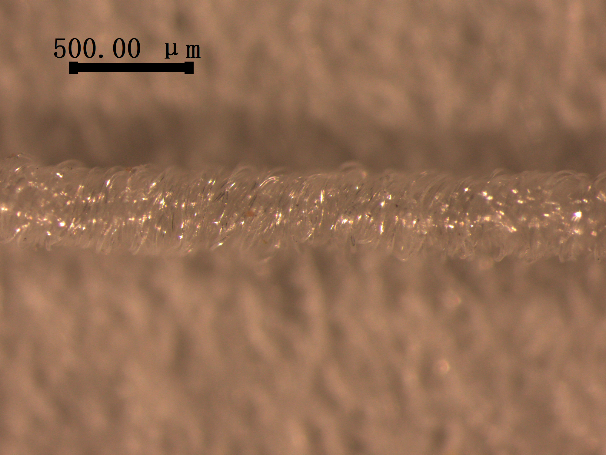


**(a)**

**(b)**

**(c)**

**(d)**

**(e)**

**(f)**

**Top View**

**Bottom View**

Figure S1. Microscopic images of (a – b) pristine yarn, (c – d) GO coated yarn, and (e – f) chemically reduced graphene yarn (GYS). Compared with white/clear appearance of the pristine yarn, the effective GO coating has been proved by the uniform grey/somber color throughout the yarn surface both on top and bottom. After the chemical-reduction treatment by immersing the yarn in 90 ⁰C pre-heated 0.06 mmol/L sodium hydrosulﬁte solution for 150 min, the rGO layer equally shows very uniform coating quality with a much darker appearance without any sign of detachment.


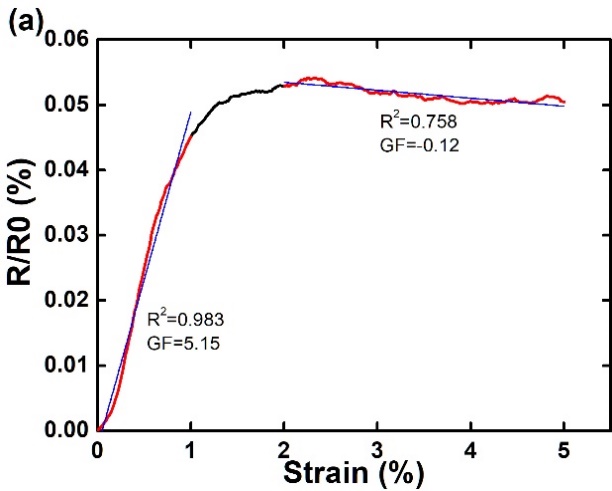

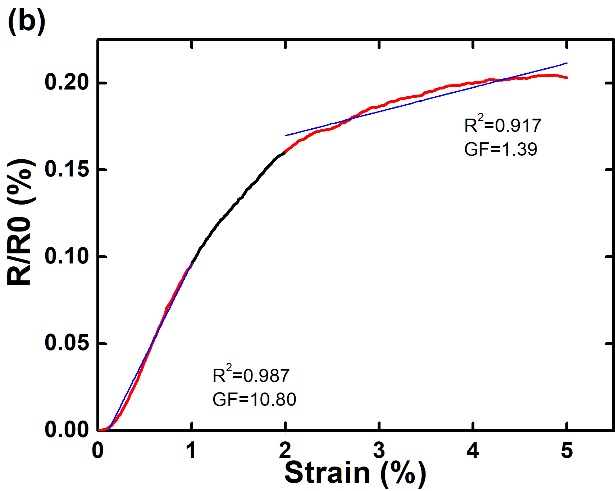

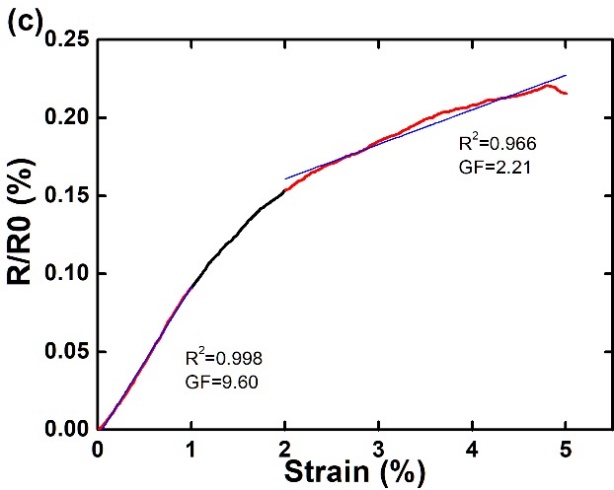

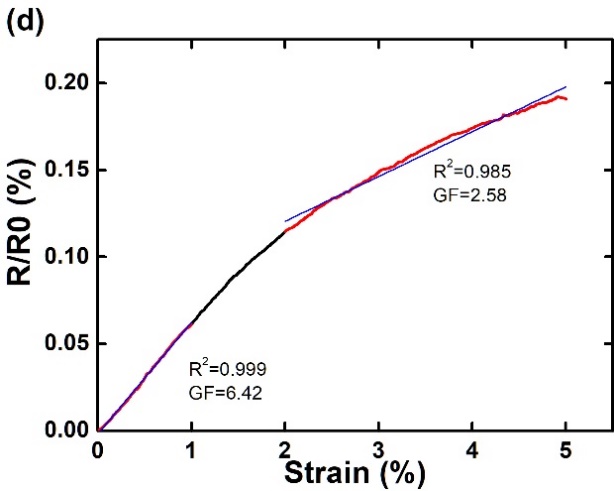


Figure S2. Linear fittings of piezoresistive response of GYS sensors processed by (a) 0%, (b) 20%, (c) 40%, and (d) 60% pre-stretching level. The pre-stretch dependent piezoresistivities are all non-linear, presenting clearly a two-regime behavior, in which the initially faster ramping of resistance change is gradually replaced by a much milder increasing. By summarizing the quantity of the coefficient of correlation (denoted R^2^), the first-regime piezoresistivies all show supreme linearity with ultra-high R^2^ values above 0.98. Comparatively, the linearity of the second-regime piezoresistivity is dominated by a prestretch dependent behavior. As the prestretch level is increased from 0 to 60%, the R^2^ value is monotonically increased from 0.758 to 0.985.

**(a)**

**(b)**

**(c)**

**(d)**

Figure S3. (a – b) Pristine GYS subjected to (a) first and (b) last 10 electromechanical cycles in 1000-cyclic-tension test. (c – d) 30% prestretched GYS subjected to (c) first and (d) last 10 electromechanical cycles in 1000-cyclic-tension test. Both the maximum GF and averaged GF of the pristine GYS show obvious decay (~54%) from 6.30 to 2.87 and 2.04 to 1.29 after 1000 cycles, not to mention its unstable initial resistance changed from 164 to 157 kΩ. The plateau signal existed in the pristine sensor once again confirms its limitation for large strain monitoring. Comparatively, the pre-stretched GYS shows much improved durability and large-strain performance. The gauge sensitivity slightly decays (~19%) from 11.77 (3.96) to 9.38 (3.28) for the maximum (averaged) GF with highly consistent initial resistance from 181 to 180 kΩ.

**(a)**

**(b)**


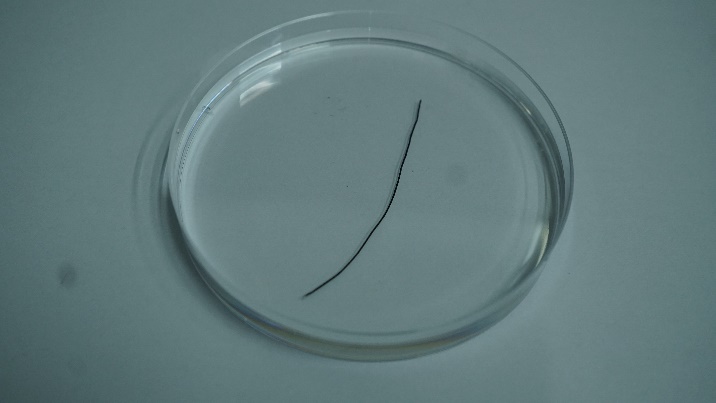

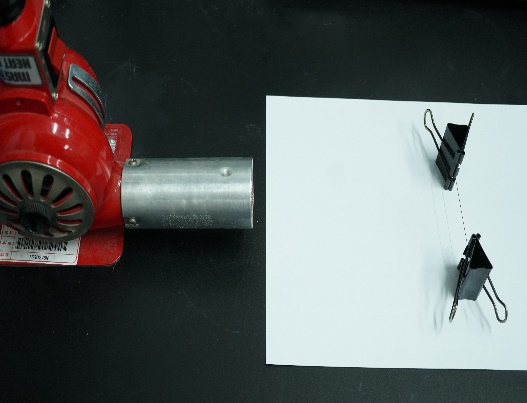


**(c)**

**(d)**

**5 cycles**

Figure S4. Photographs of (a) immersing and (b) drying process of a 30% prestretched GYS specimen; Variation of real-time resistance of the GYS subjected to 10 cyclic tensile tests (c) before and (d) after a five-cycled immersion-drying process. The initial resistance is slightly increased from 135.03 kΩ to 138.18 kΩ after the wash treatment. The gauge sensitivity i.e. GF of the specimen almost keeps the same, reading 12.82 (4.75) and 12.51 (4.32) for maximum (averaged) GF before and after the immersion-drying process.


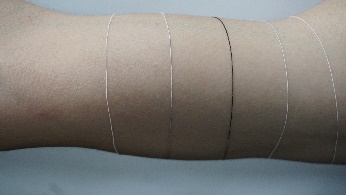

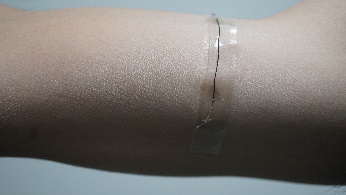

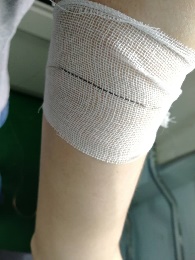


**(a)**

**(b)**

**(c)**

**(d)**

**(e)**

**(f)**

Figure S5. Photographs of (a) direct winded, (b) PDMS packaged, and (c) fabric braided GYS sensors on the upper arm. (d – f) The corresponding electrical signal for monitoring contracted & relaxed states of biceps. By considering relaxed state as the initial stage, the resistance change of contracted state i.e. the sensitivity of direct winding (37.84%) is considerably higher than PDMS packaging (17.96%) and fabric braiding (7.09%). The winding process also shows almost instant resistance increase with better stability.

**(a)**

**(b)**

**(c)**

**(d)**

**(e)**

**(f)**

Figure S6. The resistance variation of GYS under loading rate of (a) 5 mm/min, (b) 10 mm/min, (c) 20 mm/min, (d) 40 mm/min, (e) 80 mm/min. (f) The sensitivity variation of GYS at different loading rates. In terms of frequency, they correspond to 0.02 Hz, 0.04 Hz, 0.08 Hz, 0.16 Hz and 0.32 Hz). Not only is the initial resistance not affected by the loading rate, but also the gauge performance. The sensor response to the external deformation is almost instant and shows no frequency dependence, with the averaged maximum gauge sensitivity ranging from 11.20±0.9 to 12.52±1.7.
